# Supplementary material for: Genetic analysis reveals a robust and hierarchical recruitment of the LolA chaperone to the LolCDE lipoprotein transporter
Source: mBio. 2024 Jan 9;15(2):e03039-23. doi: 10.1128/mbio.03039-23 (PMC10865981; doi:10.1128/mbio.03039-23)
Supplement: Supplmental figures and tables — Figures S1-S5. Tables S1-S3. [file mbio.03039-23-s0001.pdf]

```
PDB     -------------------------------------DAASDLKSRLDKVSSFHASFTQK    23
Eco     -------------------------------------DAASDLKSRLDKVSSFHASFTQK    23
Vch     -------------------------------------APKDTLSERLAMSEGFSATFNQQ    23
Pae     ----------------------------------DDSAAVQRLTGLLNKAQTLTARFSQL    26
Lpn     ------------------------------------QTSAEVLQSKLNAIQTMTANFSQI    24
Bpe     ------------------------------------ATAQEQLDTFVATVKGATGSFKQS    24
Bps     -------------------------------------SGTEQLKAFVAKVHAAKGEFTQQ    23
Ccr     -------------------QGSTAPVPAQLSAEQKA--LLDKATAYIQGLSSAKGRFVQT    39
Bms     KAGRVAAVLGMGILGVGVAGAALAPVPVKAQGADGGIAAAQQIADHFSSARTMTGEFVQF    60
Nme     -----------------------------------QAGAVDALKQFNNDADGISGSFTQT    25
Cje     ---------------------------------------------LDLNFNTFSSNFTQI    15
Hpy     ----------------------------------KNPSTLSKEEEVLQHLQSFSAHFKQV    26
                                                              . * *

PDB     VTDG----------SGAAVQEGQGDLWVKRPNLFNWHMTQPDESILVSDGKTLWFYNPFV    73
Eco     VTDG----------SGAAVQEGQGDLWVKRPNLFNWHMTQPDESILVSDGKTLWFYNPFV    73
Vch     VLSP----------EGKVILTGNGKVDIARPSLFRWETETPDENLLVSDGTTLWHFDPFV    73
Pae     TLDG----------SGTRLQETAGQLSLKRPGLFRWHTDAPNEQLLISNGEKVWLYDPDL    76
Lpn     VK-A----------KNREVSRSSGSMALQRPGRFRWQTKDPLEQLIVADGQKMWIYDVDL    73
Bpe     TVSP----------QGATQPAQSGTFAFQRPGKFKWAVQLPYEQLIVSDGKQVFQYDPDL    74
Bps     IVKAPPKAASGAVPTVKPTDNSSGSFVFSRPGKFIWTYQKPYQQVLQADGDKLYVYDKDL    83
Nme     ------------VQSKKKTQTAHGTFKILRPGLFKWEYTKPYRQTIVGDGQTVWLYDVDL    73
Ccr     DQR---------------GTQTQGTFYLQRPGKARFAYDPPAGLLVVSNGNNVNIFDSRL    84
Bms     GPR---------------GEQTGGTFYIERPGKIRFNYNNSP-IRVISDGDSVVINNRKL    104
Cje     VKSK------------N-STLSYSGHFILSKDQAYWSYDTPSKKEIYINKNQVTIVEHDL    62
Hpy     LKNE------------K-PLVYYGVLKAKAPNWALWVYEKPLKKEIYMNDKEVVIYEPNL    73
                               .       .   :         :  :   :   :  :

PDB     EQATATWLKDATGNTPFMLIARNQSSDWQQYNIKQNG-----DDFVLTP-KASNGNLKQF    127
Eco     EQATATWLKDATGNTPFMLIARNQSSDWQQYNIKQNG-----DDFVLTP-KASNGNLKQF    127
Vch     EQVTLYRAEEALEQTPFVLLTRNKASDWDAYHVEEKG-----DVFTLTP-TALDSNQGRF    127
Pae     EQVTIQKLDQRLTQTPALLLSGDISKISESFAITYK-EGGNVVDFVLKP-KTKDTLFDTL    134
Lpn     EQVTVKNQEKGLGGTAALFLSGYDETLTHDFDVSEK-QKGKLTVFDLKS-KSAKENFQRI    131
Bpe     AQVTVRQVDQAIGTSPAAILFGAGQ-LSQAFAVSALPDRDGLQWLRAKP-RNADAGFSQV    132
Bps     NQVTERKLAGALGASPAAILFGSND-LDKNYTLRDAGEKGGIDWLEMVP-KAQDTQFQRI    141
Nme     AQVTKSSQDQAIGGSPAAILSNKTA-LESSYTLKEDGSSNGIDYVLATP-KRNNAGYQYI    131
Ccr     KTYESYPLSK----TPLNLLLAREVRLDRGVVITDVR--PLADGFTIVAQDAKRQALGRI    138
Bms     DTWDLYPLSK----TPLKLLLADRIDLGGGRLQS-VK--QEPDMTTLVLGDKSVFGDSKI    157
Cje     EQVIFSHLDNIPNLN--EIFKKASLI----------------DKDKLVA-KYDN---INY    100
Hpy     FQATITPLKDKTDFF--TILKRLKKQ----------------DDGSFKT-TINK---TTY    111
                          ::

PDB     TINVGRDGT--IHQFSAVEQDDQRSSYQLKSQQNG-AVDAAKFTFTP-PQGVTVDDQRK    182
Eco     TINVGRDGT--IHQFSAVEQDDQRSSYQLKSQQNG-AVDAAKFTFTP-PQGVTVDDQRK    182
Vch     QITISEKGV--VQGFKVIEQDGQQSEFTFSKVKQQ-KPNASVFNYKV-PKGVEVDDQRN    182
Pae     RLSF-RSGK--VNDMQMIDGVGQRTNILFFDVKMNEALDAKQFTFDV-PPGVDVIQE--    187
Lpn     KLIF-SQST--LIGLELYDQLGQITDVKLVQIKSNPKLPAKLFQFKP-PKGVDVVKQ--    184
Bpe     DIGL-RDNQ--PARIELVDAFGQTTRVELSNLLPG-AVPASEFQFTP-PQGVDVVKM--    184
Bps     GIGF-RNGM--LAAMELHDVFGNVTLLTFTNIQTNPPLKADQFKFVV-PKGADVVTG--    194
Nme     RIGF-KGGN--LAAMQLKDSFGNQTSISFGGLNTNPQLSRGAFKFTP-PKGVDVLSN--    184
Ccr     SIDF-SNGLVGLMGWTVTDIKGGQIRVRLSDFAETADLDPKLFVLTD-PRRKVGKP---    192
Bms     TMMF-DPKSYDLKQWTITDAQKLDTTVMIFNVRTGVRFTNDMFKIDY-QRIAMKRKGQ-    213
Cje     TIKL-NQEQ--IQSISYKDEFENDVIINLNNQIKNPKINSDVFKAKI-PQNYDIVR---    152
Hpy     RLVF-KDGK--PFSLEFKDGMNNLVTITFSQAEINPTIANEIFVFKPKDENIDIVRQ--    165
         : .              :         :             *
```

Fig. S1. Predicted LolA sites that make polar contacts with the LolC Pad. Aligned mature LolA protein sequences with yellow highlighted residues that are predicted by AlphaFold2 Multimer to make polar contacts to their native LolC or LolF. Blue highlighting indicates low confidence prediction. PDB denotes structure 6F3Z. LolA sequences are from *Escherichia coli* MG1655 (*Eco*), *Vibrio cholerae* O1 El Tor N16961 (*Vch*),

Pseudomonas aeruginosa PAO1 (*Pae*), *Legionella pneumophila* subsp. pneumophila Philadelphia 1 (*Lpn*), *Bordetella pertussis* Tohama I (*Bpe*), *Burkholderia pseudomallei* K96243 (*Bps*), *Neisseria meningitidis* MC58 (*Nme*), *Caulobacter vibrioides* CB15 (*Ccr*), *Brucella suis* 1330 (Bms), *Campylobacter jejuni* subsp. jejuni NCTC 11168 (*Cje*), *Helicobacter pylori* 26695 (*Hpy*). Signal peptide processed mature LolA sequences were predicted with SignalP-6.0. Alignments were made with Clustal Omega.

```
                                          |E.coli Hook|
PDB VKQTDLEPGKYN---VILGEQLASQLGVNRGDQIRVMVPSASQFTPMGRIPSQRLFNVIG 188
Eco VKQTDLEPGKYN---VILGEQLASQLGVNRGDQIRVMVPSASQFTPMGRIPSQRLFNVIG 188
Vch GRVTALQAGEYQ---LFLGHLLARSLNVTVGDKVRLMVTEASQFTPLGRLPSQRNFTVAG 193
Pae GSLDDLKPGEFG---IVLGEITARRFHVNVGDKLTLIVPEAT-SAPGGITPRMQRFTIVA 209
Lpn GNMSNLK--HFG---IILGKGLADSLGVMIGDKVTIMIPQAT-VTPAGMIPRFKRFTVVG 225
Bpe GKLSDLVAGGFG---AVLGSDLADGLGVKTGDTVLMLAPQGS-ISPAGFAPRMRQFTVVG 187
Bps GALTALAPGQFG---IVLGNALAGNLGVGVGDKVTLVAPEGT-ITPAGMMPRLKQFTVVG 194
Nme GKFEDLIPGEFD---IILGVGLAEALGAEVGNKVTVITPEGN-VTPAGVVPRLKQFTVVG 190
Ccr GSMEGFGQGEYGGDIVLIGERMAQTLGVQPGDPITIISPSGP-ATAFGSSTREKNYIVGG 202
Bms GTLKGFDHS----GGVAIGTRMAENLGLSVGDTLRVISPDGD-VTPFGVNPRVKAYPIVA 198
Cje ENL-----SGFD---ILVGSALTDEFGLHKNDKLSLIFSNLN-PSGFSLVPQTKRFDVKA 186
Hpy INENDLFKNPFN---LIVGKSLRYSLNLDLNQKADLFFTELE-PTGLTLSPIMKRFTIKG 192
                     :*      :    .:   :.  .    :        : : : .
```

Fig. S2. LolC/LolF Pad sites predicted to make polar contacts with LolA. Aligned mature LolC or LolF protein sequences from the region spanning the Pad. Yellow highlighted residues are predicted by AlphaFold2 Multimer to make polar contacts to their native LolA. Blue highlighting indicates low confidence prediction. PDB denotes structure 6F3Z. LolC sequences are from *Escherichia coli* MG1655 (*Eco*), *Vibrio cholerae* O1 El Tor N16961 (*Vch*), *Pseudomonas aeruginosa* PAO1 (*Pae*). LolF sequences are from *Legionella pneumophila* subsp. pneumophila Philadelphia 1 (*Lpn*), *Bordetella pertussis* Tohama I (*Bpe*), *Burkholderia pseudomallei* K96243 (*Bps*), *Neisseria meningitidis* MC58 (*Nme*), *Caulobacter vibrioides* CB15 (*Ccr*), *Brucella suis* 1330 (Bms), *Campylobacter jejuni* subsp. jejuni NCTC 11168 (*Cje*), *Helicobacter pylori* 26695 (*Hpy*). Alignments were made with Clustal Omega.

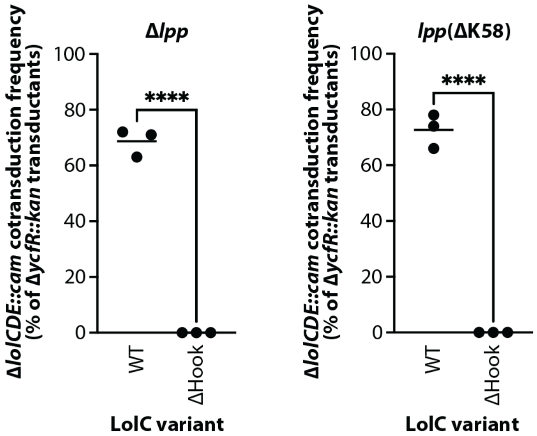

Figure S3. The LolC Hook is essential even when Lpp is detoxified or entirely absent. Genetic linkage between Δ*ycfR*::*kan* and Δ*lolCDE*::*cam* when introduced into *E. coli* mutants either lacking *lpp* or the Lpp K58 residue and carrying pBAD18::*lolCDE* plasmids encoding *lolC*(ΔHook) mutations. Three independent transductions were performed and 100 transductants from each experiment where tested (n=100 per data point); statistically significant results were found by one-way Anova (**** indicates $P<0.0001$).

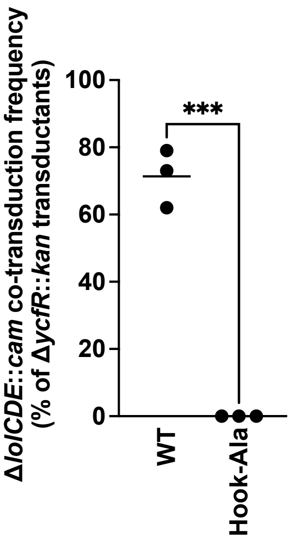

Figure S4. Plasmid expressed *lolC*(Hook-Ala) is unable to complement loss of chromosomal *lolC* even in Δ*lpp E. coli.* Genetic linkage between Δ*ycfR*::*kan* and Δ*lolCDE*::*cam* when introduced into *E. coli* mutants lacking *lpp* and carrying pBAD18::*lolCDE* plasmids encoding *lolC*(Hook-Ala) or *lolC*$^{+}$. . Three independent transductions were performed and 100 transductants from each experiment where tested (n=100 per data point); statistically significant results were found by unpaired t-test (**** indicates $P<0.0001$).

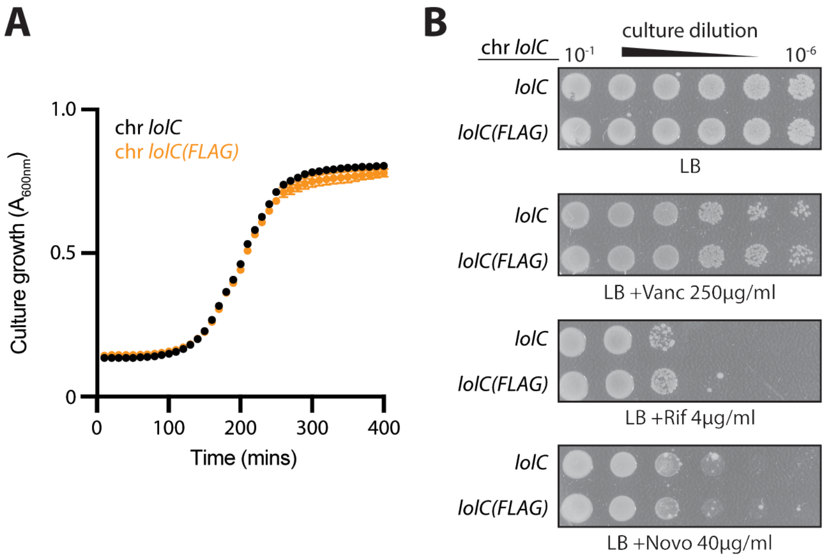

Figure S5. Appending an N-terminal FLAG tag to LolC does not appreciably affect LolC function. DNA sequence encoding an N-terminal FLAG tag was introduced into the native *lolC* locus. **(A)** Growth of a chromosomal *lolC(FLAG)* or wildtype *lolC*+ was assessed at 37°C by measuring culture turbidity (A_{600nm}) over time. **(B)** OM antibiotic barrier function was assessed by testing antibiotic sensitivity at 37°C with the antibiotics indicated.

Table S1: Strains used in this study

Table S2 Plasmids used in this study

Table S3: Oligonucleotides used in this study

Table S1. Strains used in this study

Name	Genotype	Reference
MC4100	MC4100 F- {[araD139]B/r Δ(argF-lac)169 λ- e14- flhD5301 Δ(fruK-yeiR)725(fruA25) relA1 rpsL150 rbsR22 thi-1 Δ(fimB-fimE)632(::IS1) deoC1}	(1)
NR756	MC4100 Ara^+	(2)
DY378	W3110 λcI857 Δ(*cro-bioA*)	(3)
KL351	MC4100 Ara^+ [pBAD18::*lolCDE*]	(4)
KL379	MC4100 Ara^+ [pBAD18::*lolC*(ΔHook)*DE*]	This study
KM999	MC4100 Ara^+ [pBAD18::*lolC*(M175R)*DE*]	This study
KL524	MC4100 Ara^+ [pBAD18::*lolC*(S168A)*DE*]	This study
KL515	MC4100 Ara^+ [pBAD18::*lolC*(S168D)*DE*]	This study
KL549	MC4100 Ara^+ [pBAD18::*lolC*(S168R)*DE*]	This study
KL540	MC4100 Ara^+ [pBAD18::*lolC*(S170A)*DE*]	This study
KL552	MC4100 Ara^+ [pBAD18::*lolC*(S170D)*DE*]	This study
KL527	MC4100 Ara^+ [pBAD18::*lolC*(S170R)*DE*]	This study
KL521	MC4100 Ara^+ [pBAD18::*lolC*(Q171A)*DE*]	This study
KL390	MC4100 Ara^+ [pBAD18::*lolC*(T173A)*DE*]	This study
KL518	MC4100 Ara^+ [pBAD18::*lolC*(R177A)*DE*]	This study
KL530	MC4100 Ara^+ [pBAD18::*lolC*(R177D)*DE*]	This study
KL460	MC4100 Ara^+ [pBAD18::*lolC*(I178A)*DE*]	This study
MG3437	MC4100 Ara^+ Δ*ycfR*::*kan* Δ*lolCDE*::*cam*	This study
KL466	MC4100 Ara^+ Δ*lolCDE*::*cam* [pBAD18::*lolCDE*]	This study
KM1001	MC4100 Ara^+ Δ*lolCDE*::*cam* [pBAD18::*lolC*(M175R)*DE*]	This study
KL538	MC4100 Ara^+ Δ*lolCDE*::*cam* [pBAD18::*lolC*(S168A)*DE*]	This study
KL534	MC4100 Ara^+ Δ*lolCDE*::*cam* [pBAD18::*lolC*(S168D)*DE*]	This study
KL567	MC4100 Ara^+ Δ*lolCDE*::*cam* [pBAD18::*lolC*(S168R)*DE*]	This study
KL565	MC4100 Ara^+ Δ*lolCDE*::*cam* [pBAD18::*lolC*(S170A)*DE*]	This study
KL569	MC4100 Ara^+ Δ*lolCDE*::*cam* [pBAD18::*lolC*(S170D)*DE*]	This study
KL558	MC4100 Ara^+ Δ*lolCDE*::*cam* [pBAD18::*lolC*(S170R)*DE*]	This study
KL537	MC4100 Ara^+ Δ*lolCDE*::cam [pBAD18::*lolC*(Q171A)*DE*]	This study
KL463	MC4100 Ara^+ Δ*lolCDE*::cam [pBAD18::*lolC*(T173A)*DE*]	This study
KL535	MC4100 Ara^+ Δ*lolCDE*::cam [pBAD18::*lolC*(R177A)*DE*]	This study
KL561	MC4100 Ara^+ Δ*lolCDE*::cam [pBAD18::*lolC*(R177D)*DE*]	This study
KL493	MC4100 Ara^+ Δ*lolCDE*::cam [pBAD18::*lolC*(I178A)*DE*]	This study
KL980	MC4100 Ara^+ [pBAD18::FLAG-*lolCDE*]	This study
KL1178	MC4100 Ara^+ [pBAD18::FLAG-*lolC*(ΔHook)*DE*]	This study
KL1237	MC4100 Ara^+ [pBAD18::FLAG-*lolC*(ΔM175R)*DE*]	This study
MG4583	MC4100 Ara^+ [pBAD18::FLAG-*lolCDE*]	This study
KL1272	MC4100 Ara^+ [pBAD18::*lolC*(R163A)*DE*]	This study
KM987	MC4100 Ara^+ [pBAD18::*lolC*(Q181A)*DE*]	This study
KM988	MC4100 Ara^+ [pBAD18::*lolC*(R163A Q181A)*DE*]	This study
KM989	MC4100 Ara^+ [pBAD18::*lolC*(R182A)*DE*]	This study
KM990	MC4100 Ara^+ [pBAD18::*lolC*(R163A R182A)*DE*]	This study
KM991	MC4100 Ara^+ [pBAD18::*lolC*(Q181A R182A)*DE*]	This study
KM992	MC4100 Ara^+ [pBAD18::*lolC*(R163A Q181A R182A)*DE*]	This study

KM799	MC4100 Ara⁺ Δ*lolCDE*::cam [pBAD18::*lolCDE*]	This study
KM993	MC4100 Ara⁺ Δ*lolCDE*::cam [pBAD18::*lolC*(R163A)*DE*]	This study
KL537	MC4100 Ara⁺ Δ*lolCDE*::cam [pBAD18::*lolC*(Q181A)*DE*]	This study
KM994	MC4100 Ara⁺ Δ*lolCDE*::cam [pBAD18::*lolC*(R163A Q181A)*DE*]	This study
KL464	MC4100 Ara⁺ Δ*lolCDE*::cam [pBAD18::*lolC*(R182A)*DE*]	This study
KM995	MC4100 Ara⁺ Δ*lolCDE*::cam [pBAD18::*lolC*(R163A R182A)*DE*]	This study
KM996	MC4100 Ara⁺ [pBAD18::*lolC*(Q181A R182A)*DE*]	This study
KM997	MC4100 Ara⁺ [pBAD18::*lolC*(R163A Q181A R182A)*DE*]	This study
KM1002	MC4100 Ara⁺ *zce-726*::Tn*10 lolC+*	This study
MG4644	MC4100 Ara⁺ *zce-726*::Tn*10 FLAG-lolC+*	This study
KM1003	MC4100 Ara⁺ *zce-726*::Tn*10* FLAG-*lolC*(R163A)	This study
KM1004	MC4100 Ara⁺ *zce-726*::Tn*10* FLAG-*lolC*(Q181A R182A)	This study
KM998	MC4100 Ara⁺ *zce-726*::Tn*10* FLAG-*lolC*(R163A Q181A R182A) [pBAD18::*lolCDE*]	This study
KM1005	MC4100 Ara⁺ Δ*ynhG lpp*(ΔK58) *zce-726*::Tn*10* FLAG-*lolC*	This study
KM1006	MC4100 Ara⁺ Δ*ynhG lpp*(ΔK58) *zce-726*::Tn*10* FLAG- *lolC*(R163A)	This study
KM1007	MC4100 Ara⁺ Δ*ynhG lpp*(ΔK58) *zce-726*::Tn*10* FLAG- *lolC*(Q181A R182A)	This study
KM1008	MC4100 Ara⁺ Δ*ynhG lpp*(ΔK58) *zce-726*::Tn*10* FLAG-*lolC* (R163A Q181A R182A)	This study
KM1014	MC4100 Ara⁺ *zce-726*::Tn*10* FLAG-*lolC*(M175R)	This study
KM1015	MC4100 Ara⁺ Δ*ynhG lpp*(ΔK58) *zce-726*::Tn*10* FLAG-*lolC*(M175R)	This study
KM1017	MC4100 Ara⁺ Δ*lpp* [pBAD18::*lolC*(ΔHook)*DE*]	This study
MG4726	MC4100 Ara⁺ [pBAD18::*lolC*(T173A M175R I178A)*DE*]	This study
MG4702	MC4100 Ara⁺ [pBAD18::FLAG-*lolC*(T173A M175R I178A)*DE*]	This study
MG4727	MC4100 Ara⁺ [pBAD18::*lolC*(Hook-Ala)*DE*]	This study
MG4705	MC4100 Ara⁺ [pBAD18::FLAG-*lolC*(Hook-Ala)*DE*]	This study
MG4728	MC4100 Ara⁺ Δ*lpp* [pBAD18::-*lolC*(Hook-Ala)*DE*]	This study
KM1018	MC4100 Ara⁺ *lpp*(ΔK58) [pBAD18::*lolC*(ΔHook)*DE*]	This study
MG4595	DY378 Δ*lpp*::*kan* Δ*lolCDE*::*cam* [pBAD18::*lolCDE*]	This study
MG2502	MC4100 Ara⁺ Δ*ynhG lpp*(ΔK58) *zce-726*::Tn*10*	This study
MG4655	MC4100 Ara⁺ Δ*ynhG lpp*(ΔK58) *zce-726*::Tn*10 lolC* (R163A Q181A R182A)	This study

1. Casadaban MJ. 1976. Transposition and fusion of the lac genes to selected promoters in Escherichia coli using bacteriophage lambda and Mu. J Mol Biology 104:541–555.
2. Button JE, Silhavy TJ, Ruiz N. 2007. A Suppressor of Cell Death Caused by the Loss of σ E Downregulates Extracytoplasmic Stress Responses and Outer Membrane Vesicle Production in Escherichia coli. J Bacteriol 189:1523–1530.
3. Yu D, Ellis HM, Lee E-C, Jenkins NA, Copeland NG, Court DL. 2000. An efficient recombination system for chromosome engineering in Escherichia coli. Proc National Acad Sci 97:5978–5983.
4. Grabowicz M, Silhavy TJ. 2017. Redefining the essential trafficking pathway for outer membrane lipoproteins. Proc National Acad Sci 114:4769–4774.

Table S2. Plasmids used in this study

Name	Description	Reference
pBAD18	Arabinose inducible, Amp^R, cloning vector	(1)
pABD18::lolCDE	*lolCDE* cloned to be arabinose inducible, Amp^R	(2)

1. Guzman LM, Belin D, Carson MJ, Beckwith J. 1995. Tight regulation, modulation, and high-level expression by vectors containing the arabinose PBAD promoter. J Bacteriol 177:4121–4130.
2. Grabowicz M, Silhavy TJ. 2017. Redefining the essential trafficking pathway for outer membrane lipoproteins. Proc National Acad Sci 114:4769–4774.

Table S3. Oligonucleotides used in this study

Name	Sequence	Use
deltahookLolC _F	ccaagccagcgcctgttc	*lolC* mutagenesis
deltahookLolC _R	tggtaccatcacgcggatttg	*lolC* mutagenesis
hook-Ala-LolC _F	gcgccggcggcggctgctccaagccagcgc	*lolC* mutagenesis
hook-Ala-LolC _R	ggccgcggcggcagctggtaccatcacgcg	*lolC* mutagenesis
T173A M175R I178A LolC _F	gcgccgcgtgggcgtgctccaagccagcgc	*lolC* mutagenesis
T173A M175R I178A LolC _R	gaactggctggcagat	*lolC* mutagenesis
R163A_LolC_ R	ccgcgattaacgcctagc	*lolC* mutagenesis
R163A_LolC_ F	tgatcaaatcgccgtgatggtaccatctgc	*lolC* mutagenesis
M175R_LolC_ F	gttcacgccgcgtgggcgtattccaagc	*lolC* mutagenesis
M175R_LolC_ R	tggctggcagatggtacc	*lolC* mutagenesis
T173A_LolC_ F	cagccagttcgctccgatggggc	*lolC* mutagenesis
T173A_LolC_ R	gcagatggtaccatcacgc	*lolC* mutagenesis
I178A_LolC_F	gatggggcgtgctccaagccagcg	*lolC* mutagenesis
R182A_LolC_ F	tccaagccaggccctgttcaatgtgattggc	*lolC* mutagenesis
182A_LolC_R	atacgccccatcggcgtg	*lolC* mutagenesis
I178A_LolC_R	ggcgtgaactggctggca	*lolC* mutagenesis
Q181A_LolC_ R	tattccaagcgcgcgcctgttcaatgtgattggc	*lolC* mutagenesis
Q181A_LolC_ F	cgccccatcggcgtgaac	*lolC* mutagenesis
Q171A_LolC_ F	atctgccagcgcgttcacgccgatg	*lolC* mutagenesis
Q171A_LolC_ R	ggtaccatcacgcggatt	*lolC* mutagenesis
R177A_LolC_ R	gtgaactggctggcagat	*lolC* mutagenesis
R177A_LolC_ F	gccgatgggggctattccaagccag	*lolC* mutagenesis
F172A_LolC_ R	gatggtaccatcacgcgg	*lolC* mutagenesis
F172A_LolC_F	tgccagccaggccacgccgatgg	*lolC* mutagenesis
S168A_F	gcggccagccagttcac	*lolC* mutagenesis
S168_R	tggtaccatcacgcggatttg	*lolC* mutagenesis
S168D_F	gatgccagccagttcacg	*lolC* mutagenesis
S168R_F	cgtgccagccagttcac	*lolC* mutagenesis
S170A_F	gcgcagttcacgccgatg	*lolC* mutagenesis

S170_R	ggcagatggtaccatcac	*lolC* mutagenesis
S170D_F	gaccagttcacgccgatg	*lolC* mutagenesis
S170R_F	cgtcagttcacgccgatg	*lolC* mutagenesis
Q171A_F	gcgttcacgccgatgggg	*lolC* mutagenesis
Q171_R	gctggcagatggtaccatcac	*lolC* mutagenesis
R177A_F	gctattccaagccagcgc	*lolC* mutagenesis
R177_R	ccccatcggcgtgaactg	*lolC* mutagenesis
R177D_F	gatattccaagccagcgc	*lolC* mutagenesis
F172_R	ctggctggcagatggtac	*lolC* mutagenesis
F172S_F	tctacgccgatggggc	*lolC* mutagenesis
F172R_R	cgtacgccgatgggg	*lolC* mutagenesis
F172D_F	gatacgccgatggggcg	*lolC* mutagenesis
LolC_1812_R	gcttggaatacgcccc	*lolC* mutagenesis
LolC_Q181A_F	gcgcgcctgttcaatgtgatt	*lolC* mutagenesis
LolC_R182A_F	caggcgctgttcaatgtgattggcactttc	*lolC* mutagenesis
LolC_QR181AA_F	gcggcgctgttcaatgtgattggcactttc	*lolC* mutagenesis
rec_p18lolCDE_fwd	gattgatttacgggggcttttcagattagccctgacgatcacttacagttcagacgtttacccat cttgctttcgcttatatactcgtgtctttgctacagcaacc	Recombineering template
rec_p18lolCDE_rev	ctgcaactgccgaccgctatcaaacacgccaagcgcaatttttgttccaccaatatcaaacccgt aatacattgccgctccttgttttaatgtactgcctttactgg	Recombineering template
KM178LolC_N -FLAG_F	atggactacaaagacgatgacgacaagtaccaacctgt cgctctatt	FLAG tagging
KM177LolC_N -tag_R	gaaatccgtctggttgctgtag	FLAG tagging
